# Solubility and stability enhancement of ethanol in diesel fuel by using tri-n-butyl phosphate as a new surfactant for CI engine

**DOI:** 10.1038/s41598-023-45252-7

**Published:** 2023-10-20

**Authors:** Mostafa M. El-Sheekh, Aya A. El-Nagar, Medhat ElKelawy, Hagar Alm-Eldin Bastawissi

**Affiliations:** 1 Botany Department, Faculty of Science, Tanta University, Tanta, 31527 Egypt; 2 Microbial Biotechnology Department, Genetic Engineering and Biotechnology Research Institute, University of Sadat City, Sadat City, Egypt; 3 Mechanical Power Engineering Departments, Faculty of Engineering, Tanta University, Tanta, Egypt

**Keywords:** Climate sciences, Environmental sciences, Energy science and technology, Materials science

## Abstract

Nowadays, researchers are very interested in improving the stability and solubility of blending diesel fuel with a high percentage of ethanol. As a result, the goal of this paper was to find a way to use the surfactant of Tri-n-butyl phosphate (TBP) substance to blend ethanol with diesel fuel to a level of 40%. Diesel fuel is mixed with ethanol in volumetric proportions of 10%, 20%, 30%, and 40%, as well as a tiny amount of TBP from 1 to 4%. The prepared blends were the subject of an experiment evaluation by fueling a direct injection diesel engine. This engine is a water-cooled, commercial diesel engine, single cylinder, and four-stroke with 12 kW maximum power. The four blends were evaluated as clean fuel mixtures of 10% ethanol/90% diesel/1% TBP, 20% ethanol/80% diesel/2% TBP, 30% ethanol/70% diesel/3% TBP, and 40% ethanol/60% diesel/4% TBP. As the starting fuel, we used 100% diesel to compare the results. The engine’s output and emissions have been measured at various engine loads and constant speeds of 1500 rpm. According to the data gathered, even when the percentage of ethanol was increased to 40%, neither the base fuel nor the engine BTE changed significantly. The engine exhaust gas temperature was found to decrease slightly when the proportion of ethanol was increased. When bioethanol is increased to 40% of the base volume, it causes an increase in the combustion of unburned hydrocarbons and CO emissions. However, when the percentage of ethanol was increased from 100% diesel to the base fuel to 40%, CO_2_ emissions decreased, and O_2_ emissions slightly increased.

## Introduction

It is well recognized that a country's economic development is closely correlated with its energy use, which is highly correlated with pollution emissions. Consequently, global warming and other environmental problems are connected to these emissions^1^. The most known pollutant of the environment is caused by the combustion of fossil fuels, which is considered the primary energy source used today^2^. As a result, there is interest in discovering alternatives to fossil fuels and replacing them to ensure sustainable economic growth and meet strict pollution emission standards. As a result, using alternative fuels solves both environmental issues and worries about energy security. Biofuels are a viable alternative that can be adopted to grow the rural economy and save money in foreign exchange^3^. Examples of such fuels include methanol, ethanol, biodiesel, and gaseous fuels like methane and hydrogen^4^. These days, researchers and governments are seeking to use these kinds of fuels to be more ecologically sound, reducing the concentration of CO_2_ during the combustion process, and support sustainability because they are readily available^5–7^. However, since they typically emit fewer emissions than fossil fuels, the biggest drawback of biofuels is their performance. However, engine operation with these fuels needs in-depth research to be optimized depending on the source and its composition^8^. Animal and chicken waste fats biodiesel or novel fuel additives have been used to improve diesel engine combustion and emissions attribute^9–11^. The obtained results show that the engine BTE will improve, and the recorded emissions show a remarkable reduction in smoke, CO, and UHC emissions. However, the engine performance was improved by using fuel additive in order to increase the fuel Cetane number^12–14^. The recorder results confirmed that the unburned hydrocarbon and carbon dioxide could be reduced by 50% by increasing the fuel Cetane number. A new trend for optimizing the engine response parameters such as BTE and engine emissions with varying operating parameters has been studied by using response surface methodology^15–17^. By utilizing these techniques, the number of laboratory trails was dramatically reduced and maximum engine performance was achieved.

One excellent solution to these issues is blending ethanol with diesel fuel. Numerous studies that combined ethanol and diesel fuel discovered that because ethanol and diesel fuel are not miscible, only a minimal amount of ethanol (less than 5%) is permitted in the blending process for best outcomes^18^. Surfactant is added to the ethanol/diesel blend to raise the ethanol ratio in the mixture^19^. To stabilize combinations of ethanol and diesel, fatty acid methyl esters (FAME) could also be used as a surface-active agent^20–22^. Ten % ethanol could be used in diesel/biodiesel blend as (10% ethanol, 20%Jatropha biodiesel, and 70% diesel), In comparison to the ternary fuel (TF), it was discovered that the 20 ppm alumina nano addition (TF20) increased BTE, decreased BSEC by 7.8% and 4.93%, and decreased HC, NOx, CO, and smoke emissions by 5.69%, 9.39%, 11.24%, and 6.48%^23^. According to^24^, the ethanol content of a diesel/biodiesel combination exceeds 20%. Who tested 10% bioethanol/45% diesel/45% biodiesel and 20% bioethanol/40% diesel/40% biodiesel as novel fuel blends in a single-cylinder water-cooled direct injection diesel engine? The collected data show that adding 10% bioethanol improved the engine's BTE while reducing its NOx output. The combustion of unburned hydrocarbons and CO emission rise while bioethanol is increased to 20% of the base volume. Ethanol fuel is used as an additive to diesel and gasoline fuel for reducing engine emissions^25,26^. The mathematical formulation has been applied to study the engine energy and exergy in the case of using different fuel blends^27^. Furthermore, the nanomaterials additives were employed to improve the engine attributes and exhaust gas emissions at different engine loads^28,29^. The using of borax decahydrate and graphene additives with 100 ppm concentration in the fuel commixture will improve the engine thermal efficiency and reduce the CO and UHC emissions.

Maintaining minimal engine modifications is one of the critical objectives of using fuel blends in engines; therefore, blend stability and creating a single-phase liquid system are required^24,30^. Surfactant-free microemulsions (SFMEs) such as (propanol, butanol), *N*, *N* dimethylformamide, acetone, and tetrahydrofurfuryl alcohol can be used to increase the blend stability^31^. The emissions of unburned hydrocarbons reduced significantly under various engine load levels when^32^ applied a new alternative fuel (algal biodiesel) to the diesel/n-pentane mixture before being burned inside the diesel engine cylinder. This study discovered that Tri-n-butyl phosphate (TBP) could be added to an ethanol/diesel blend to raise the ethanol content to 40%. This amount is high, reaching 40% ethanol when combined with diesel.

**Figure 1:**
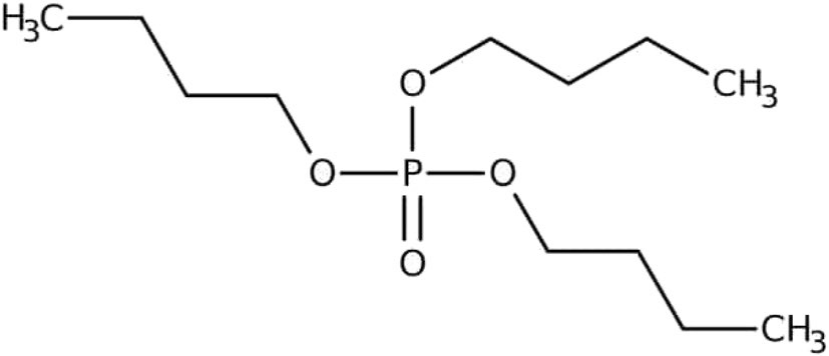
Chemical structure of TBP^33^, Formula: C_12_H_27_O_4_P, Mol. weight: 266.31.

Figure 1 shows the chemical structure of tri-n-butyl phosphate (TnBP), a typical alkyl organophosphate esters (OPEs) compound. This odourless, colourless liquid has specific uses as a plasticizer and an extractant. It is an n-butanol and phosphoric acid ester^33^. TBP is a solvent in inks, artificial resins, gums, adhesives, and concentrations of herbicides and fungicides. It is utilized as an anti-foaming ingredient in detergent solutions and other emulsions, paints, and bonds because it has no odour.

Additionally, it can be found in ethylene glycol-borax antifreeze solutions as a de-foamer. Adding TBP to oil-based lubricants strengthens the oil film and the nuclear industry^34,35^. Phosphoryl chloride and n-butanol combine to produce tributyl phosphate^35^, as presented in the following Eq. (1).

1 $$ {\text{POCl}}_{{3}} + {\text{ 3 C}}_{{4}} {\text{H}}_{{9}} {\text{OH}} \to {\text{PO}}\left( {{\text{OC}}_{{4}} {\text{H}}_{{9}} } \right){3} + {\text{3 HCl}} $$

Ethanol is a good fuel for spark-ignition engines due to its high octane number^36^. However, it's also clear that alcohols, specifically ethanol and to a much lesser amount methanol, have been considered as a substitute for diesel engines fuels^37^. The attributes of ethanol-based fuels can now be used with diesel engine technology using various methods. They can be broadly categorized into three classes: The amount of ethanol that can be utilized in this method is constrained by the onset of avoidance of flame quenching and misfire at low loads, and engine knock at high loads. (a) Ethanol fumigation to the intake air charge via carburetion or manifold injection^38^. (b) Dual injection systems are not thought to be very practical because they require an additional high-pressure injection system and a significant change in the cylinder head’s design^39^. And (c) (emulsions), ethanol, and diesel fuel blends, which require no technical engine side modifications^40^.

It is noted that when referring to stable emulsions, the terms “micro-emulsions of ethanol in diesel fuel” and “solutions of anhydrous ethanol in diesel fuel” are frequently used interchangeably (translucent). Anhydrous ethanol (200 proof) can be dissolved transparently in diesel fuel without using an emulsifying agent or so-called “surfactant,” but these solutions can only withstand 0.5% water. Afterwards, an emulsifying agent is needed in the practical circumstances of employing lower-proof ethanol (let’s say 190 proof or lower) to create the opaque macro-emulsion; if allowed to stagnate for an extended period, this could separate into the two phases. From the perspective of engine durability, blends of diesel fuel containing ethanol up to 15% (by vol.) are generally considered safe.

Although n-butanol has not been extensively tested in diesel engines, it is a powerful competitor to alcohol for usage as fuel in these engines. Because it is less hydrophilic than ethanol and has higher miscibility, higher cetane number, more excellent heating value, and lower vapour pressure than ethanol, butanol is of particular interest as a sustainable biofuel. This makes it better than ethanol for mixing with conventional diesel fuel. Therefore, employing butanol, which has qualities considerably near to diesel than ethanol fuel, solves the issues with ethanol as a diesel fuel that were discussed in the preceding part to a significant amount^41,42^.

Because butanol’s 4-carbon structure can either form a straight chain or a branched system, giving rise to diverse characteristics, butanol is a more complex alcohol (higher-chain) than ethanol. Then, depending on the position of the hydroxyl group (–OH) and the carbon chain structure, it exists as several isomers, with butanol synthesis from biomass typically producing mostly straight-chain molecules. 1-Butanol has a straight-chain structure with the hydroxyl group (–OH) at the terminal carbon and is also more commonly referred to as n-Butanol (normal butanol)^43^. The effect of n-butanol level in diesel fuel on the efficiency and emissions of a heavy-duty (DI) diesel engine was investigated In Ref.^44^. Multi-injection fuel capability was used at fixed engine speeds and loads, and exhaust gas recirculation rates were altered to maintain constant NOx emissions. Vegetable oil-fueled diesel engines' efficiency and exhaust emission attributes when mixed with oxygenated organic molecules like ethanol and n- n-butanol, are the subject of two more studies by a different research team^45,46^. Additionally, in Ref.^46^, a limited amount of n- n-butanol was utilized solely to increase the solubility of the ethanol/diesel fuel blends that were being employed.

The authors' best knowledge indicates that there needs to be more published research on the use of Tri-n-butyl phosphate as a new surfactant to enhance diesel engine ethanol/diesel fuel blends. However, its effects on the engine's performance and emissions have been thoroughly examined using a test bench consists of a single-cylinder diesel engine. This paper planned the usage of Tri-n-butyl phosphate (TBP) as a new substance in working on the dependability and solvency of ethanol/diesel mix for improving the diesel engine execution and decreasing the hurtful outflows emissions.

## Material and methods

### Blends preparation and tri-n-butyl phosphate additive to each blend

Four blends of 10% Ethanol/90% Diesel, 20% Ethanol/80% Diesel, 30% Ethanol/70%, and 40% Ethanol/60% Diesel were prepared. Different concentrations of TBP (1%, 2%, 3% & 4%) were applied to each blend to detect the best engagement that enhanced the ethanol/ diesel solubility. Diesel fuel was used as base fuel. The physical and chemical properties of diesel and ethanol fuels are listed in Table 1.

**Table 1:** The chemical and physical properties of the diesel #1 and ethanol fuel.

Characteristics/units	Diesel#1	Ethanol	Norms
The temperature of ignition ^o^C	265		ASTM D-1929–20
Viscosity (mm^2^ s^−1^)	2.39	1.2	ASTM D-445
Density (kg m^−3^)	0.825	0.789	ASTM D-4052
Point of cloud (°C)	0		ASTM D-97
Cetane index	49		ASTM D-976
Flashpoint (°C)	55	13	ASTM D-93
Heating value (MJ kg^−1^)	42.843	27	ASTM D-240
Flammability limits (%)	0.5–5.7	3.4–19.2	–
Vapour pressure at 25 °C (kPa)	0.3	17	–
Flammability limits (℃)	65–150	12–43	–
Auto-ignition ability (℃)	230	366	–
Chemical formula	C_x_H_y_	C_2_H_5_OH	–

### Test engine arrangement and uncertainty test

**Figure 2:**
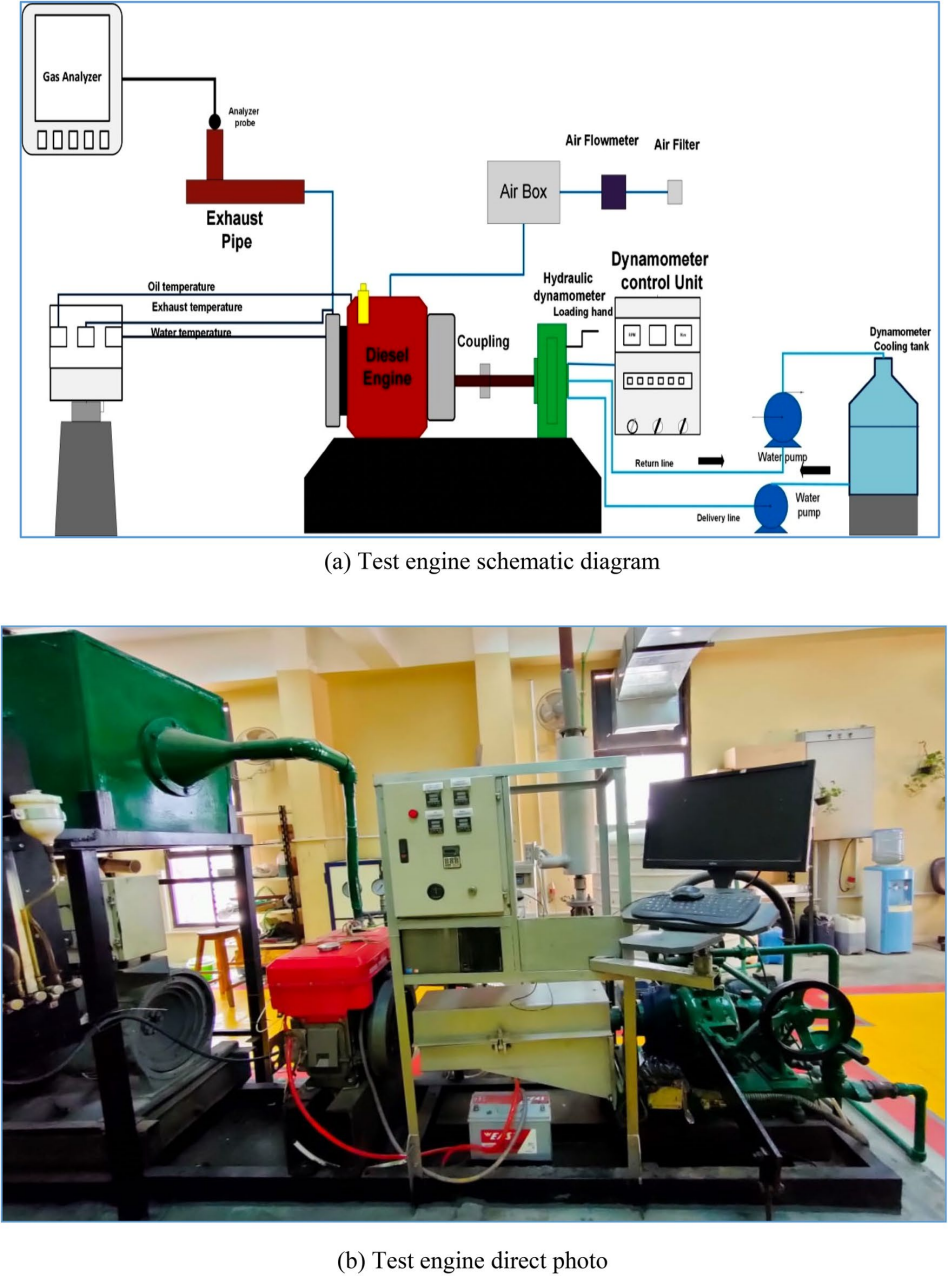
a, b shows the test engine's configuration with all the measuring arrangements, (**a**) the test bench's arrangement, and (**b**) a direct view of the measuring system.

The test engine consists of a single-cylinder direct injection diesel engine, as can be seen in Fig. 2^47,48^. The engine is connected to a hydraulic dynamometer to measure the engine's performance at different load conditions. However, the system layout is shown in Fig. 2a. In addition, the direct photo of the engine with the measuring devices is presented in Fig. 2b. The engine fuel consumption and airflow rate were measured, and the temperature of the complete five points of the engine was recorded. The k-type thermocouple is used for the temperature measurements. The tested engine is a water-cooled single cylinder with a compression ratio of 17:1 with a bore stroke dimension of 100,105 mm respectively, and the engine maximum power is 12 kW. The engine cooling system, as well as the engine value information, was listed in the table. The complete system devices, as well as the uncertainty value for each device, are listed in Tables 2 and 3. The exhaust gasses such as NO_X_, CO, CO_2_, O_2_, and UHC were recorded by using an exhaust gas analyzer^49–51^. The fuel blends were prepared outside the engine tank after adding Tri-n-butyl Phosphate as a new Surfactant into the fuel with different doses until the commixture became homogenized. At the same time, the fuel blends, solubility, and stability were tested and optimized as in the following section.

**Table 2:** Exhaust gas analyzer system data.

Gas type	m measuring range	Resolution	Error value
HC	0: 10,000	1 PPM	± 10 ppm
NOx	0: 5000	1 PPM	± 2 ppm
CO2	0:20 vol%	0.01% vol	± 0.05%
CO	0:0.1 vol%	0.01 vol%	± 0.03%

**Table 3:** Accuracy information and the key features of used devices.

Device used	Accuracy percentage	Specification
Fuel stream	± 0.075	Differential pressure type
Temp. recorder	± 1.5	Exhaust gas, Cooling air, engine block, and fresh air temperatures
Airflow stream	± 0.5	Model SLI., Make wika
Cranking	± 0.1	Make Kubler temperature, oil temperature

Motor working and actual boundaries cause some vulnerability in the exploratory outcomes. Accordingly, vulnerability examination is fundamental to guarantee the reasonable arrangement precision. Vulnerability estimations zeroed in on motor working boundaries, for example, brake power and biodiesel mixing and NOx, UHC, and BTE as motor reactions. Vulnerability computations for motor result reactions were expressed by the follows condition:

$$ {\text{w}}_{{\text{R}}} = { }\left( {\left( {\frac{{\partial {\text{R}}}}{{\partial {\text{x}}_{1} }}{\text{w}}_{1} } \right)^{2} { } + { }\left( {\frac{{\partial {\text{R}}}}{{\partial {\text{x}}_{2} }}{\text{w}}_{2} } \right)^{2} { } + { } \cdots { } + { }\left( {\frac{{\partial {\text{R}}}}{{\partial {\text{x}}_{{\text{n}}} }}{\text{w}}_{{\text{n}}} } \right)^{2} } \right)^{\frac{1}{2}} $$

Uncertainty value for biodiesel blending NOx, break power, UHC, and BTE was determined respectively. However, the total error for measuring these values was record to be 2.3%.

## Results and discussion

### Fuel blends solubility and stability

**Figure 3:**
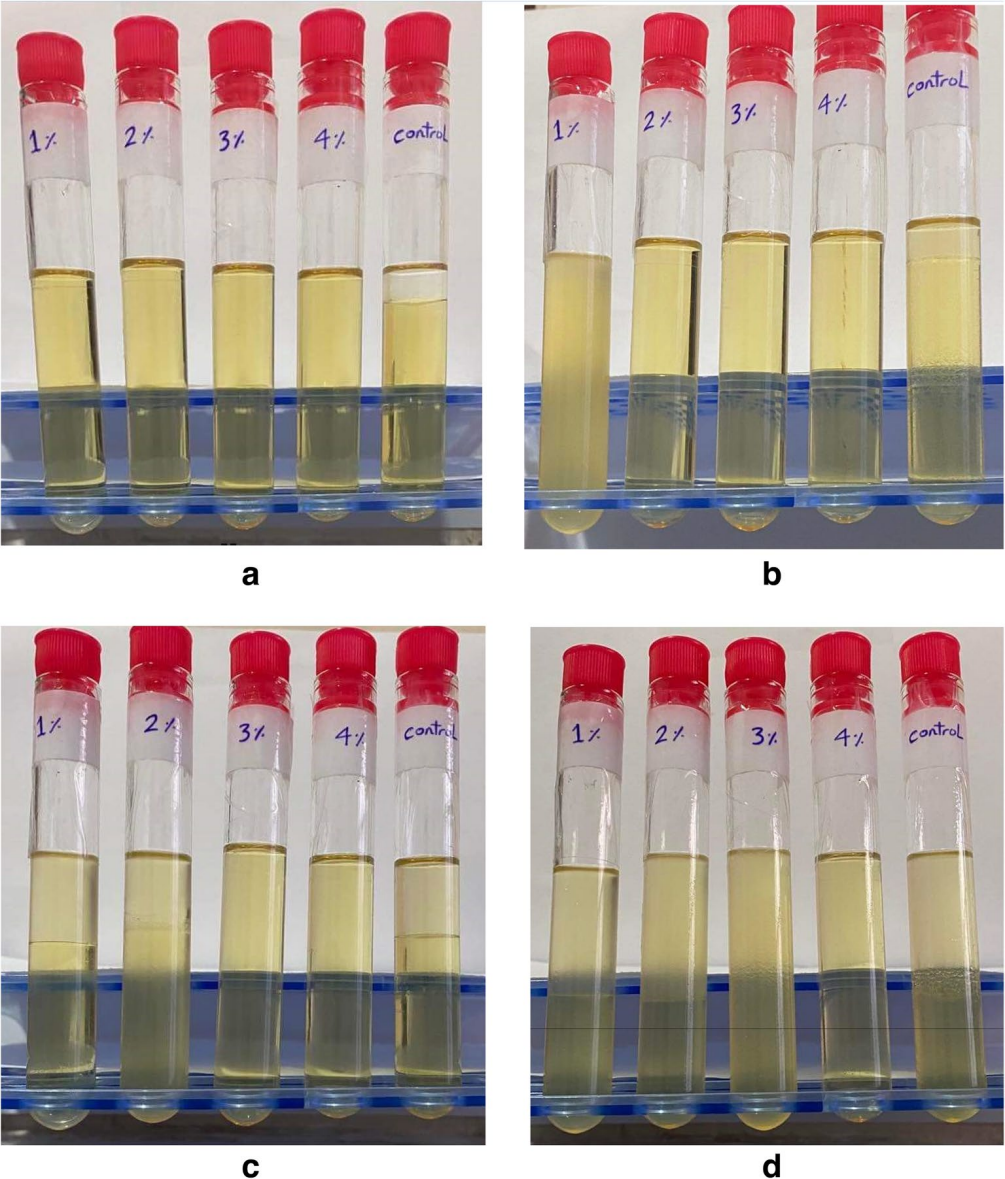
Stability of ethanol inside diesel fuel with 1, 2, 3, 4% TBP. (**a**) 10% ethanol& 90% diesel, (**b**) 20% ethanol& 80% diesel, (**c**) 30% ethanol& 70% diesel, and (**d**) 40% ethanol& 60% diesel.

The stability of four blends of 10%Ethanol/90% Diesel, 20%Ethanol/80% Diesel, 30%Ethanol/70%, and 40%Ethanol/60% Diesel was shown in Fig. 3. Without the requirement for a mixing apparatus, it was found that TBP improved the solubility and stability of the ethanol/diesel blend. This may be because n-butanol is less hydrophilic than ethanol and has higher miscibility, higher cetane number, more excellent heating value, and lower vapour pressure than ethanol, butanol is of particular interest as a sustainable biofuel. This makes it better than ethanol for mixing with traditional diesel fuel^41,42^. It was observed that increasing the ethanol percentage needs more TBP additive. (10%Ethanol/90% Diesel) blend needs 1% TBP to be stable, as in Fig. 3a. 2%, 3%, and 4% TBP were the best concentrations for (20%Ethanol/80% Diesel, 30%Ethanol/70% and 40%Ethanol/60% Diesel) blends, respectively, as in Fig. 3b–d.

### Performance and emission analysis of a diesel/ethanol/TBP-fueled DI-diesel engine

At a fixed 1500 RPM speed, the performance and emissions of DI-Diesel engines will be examined under various engine load circumstances. Four fuel blends have been used at the engine's steady-state condition. The examination of engine performance will consider how ethanol affects engine BTE, BSFC, and exhaust gas temperature as engine load varies with aid of TBP. The effect of ethanol and TBP addition on CO, CO_2_, UHCs, NOx, and O_2_ emissions at various engine loads will be reflected in the engine emissions.

#### Ethanol and TBP’s impact on performance characteristics of the engine

The experimental results of the current work illustrate the effect of increasing the ethanol dose as a renewable fuel source additive in diesel fuel on engine performance and emissions. The ethanol dose is increased up to 40% by volume. The increasing ethanol doses increase the probability of phase separation of the fuel blends. Using TBP additives as a surfactant will enhance the solubility and stability of ethanol in the base diesel fuel. However, the engine performance and emissions characteristics have been investigated at different ethanol doses with TBP additives as a surfactant while the engine load conditions were changed.

**Figure 4:**
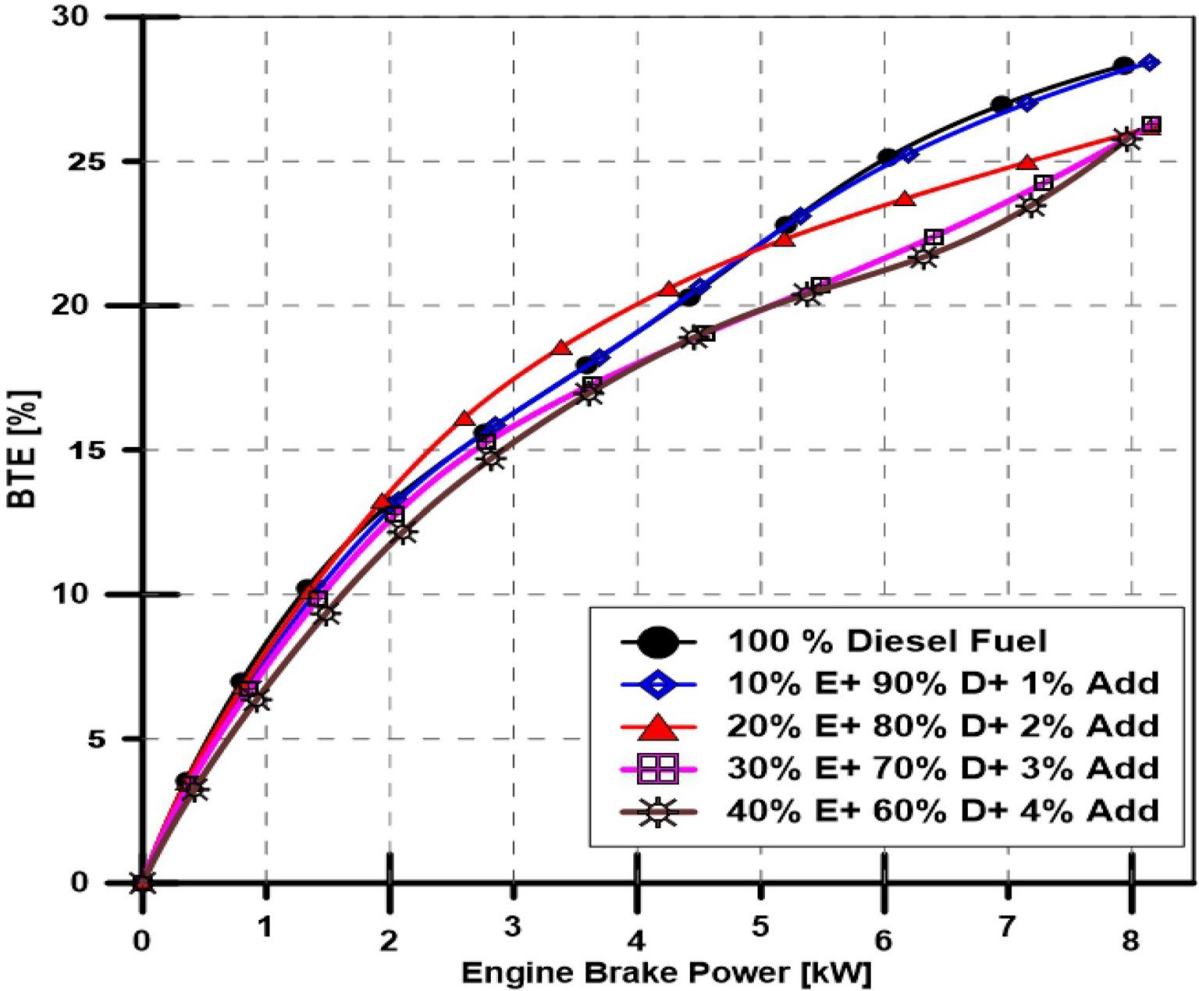
Effect of ethanol and TBP additives on engine thermal efficiency.

**Figure 5:**
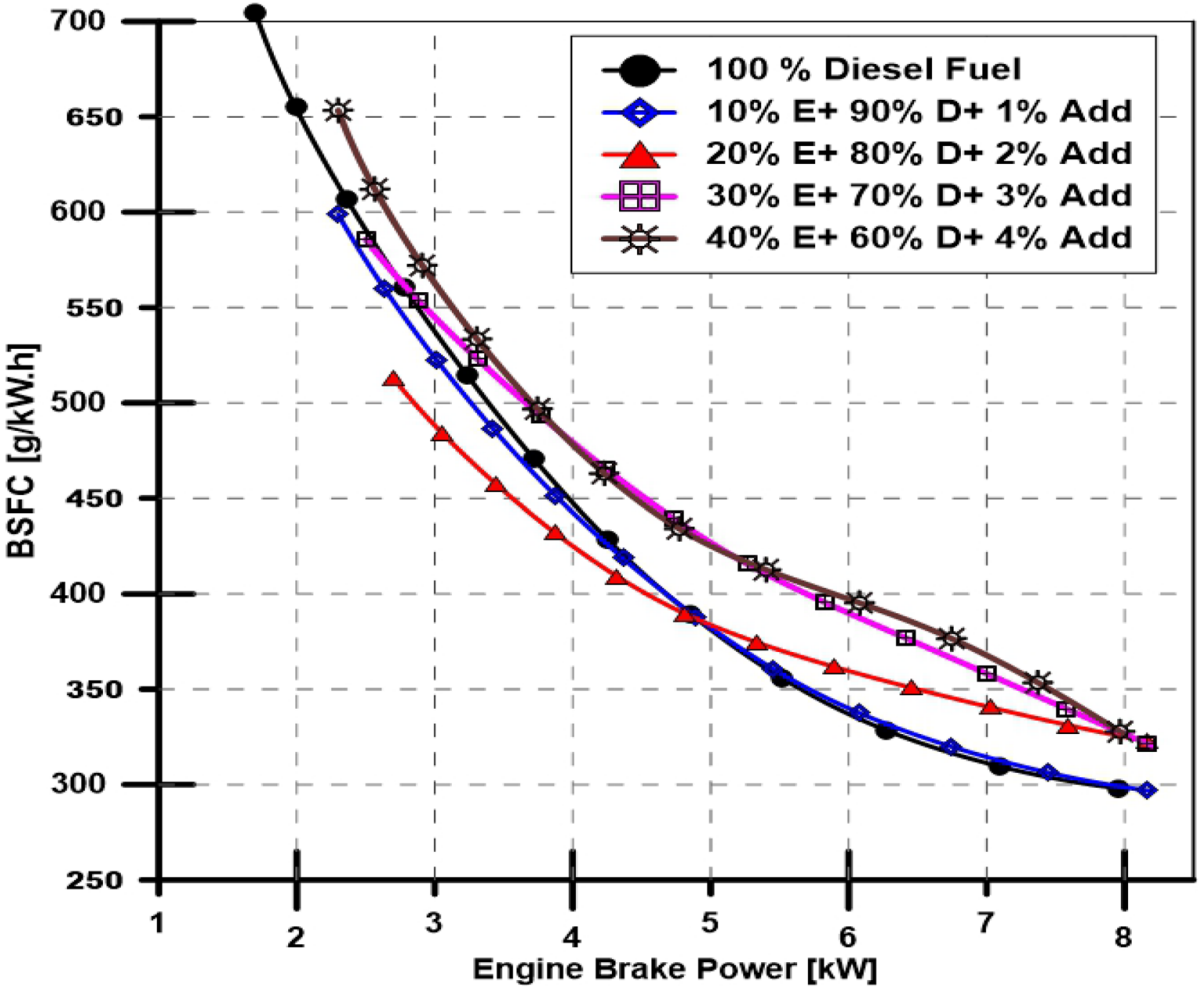
Effect of ethanol and TBP additives on engine BSFC.

**Figure 6:**
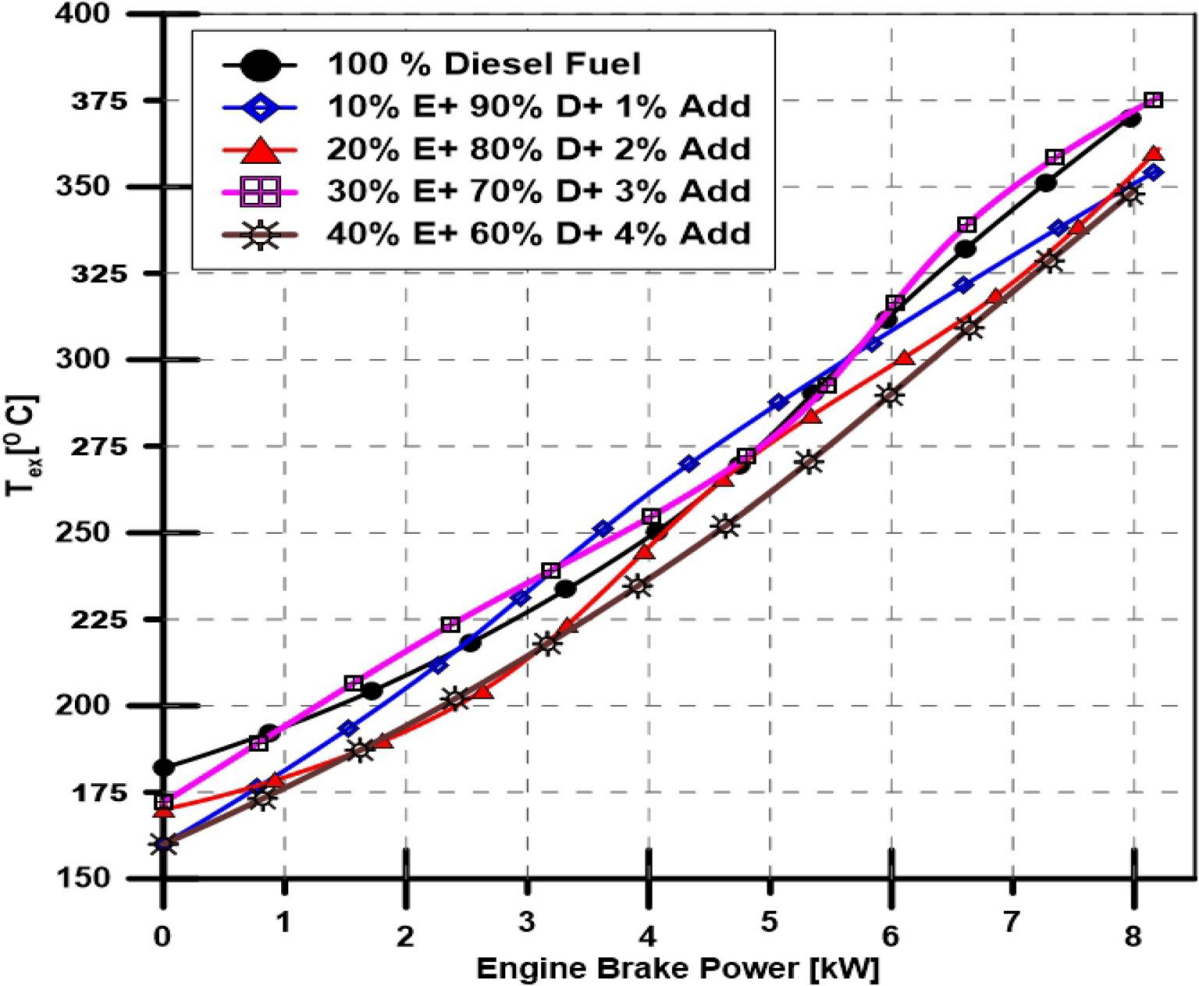
Effect of ethanol and TBP additives on the exhaust gas temperature.

A performance characteristic has been investigated by calculating the engine thermal efficiency and BSFC with varying engine power at a constant 1500-RPM speed. Figures 4 and 5 depict the engine brake thermal efficiency (BTE) and BSFC, respectively. Figure 6 illustrates the exhaust gas temperature for various engine loads and different bioethanol percentage doses. The engine's minimum and maximum obtained loads were used to vary the BSFC, which ranged from 700 to 290 g (kWh) -1—even with the ethanol proportion increased to 40%, the BSFC indicates an underwhelming rise. Additionally, as seen in Fig. 5, the engine BTE exhibits an unimpressive drop. The effects of ethanol additives in diesel fuel on engine exhaust gas temperature are added to the findings as mentioned above and are depicted in Fig. 6, which shows that increasing the percentage of ethanol causes a slight decrease in engine exhaust gas temperature.

#### Ethanol and TBP's impact on engine CO emission

The fuel combustion process starts from fuel charging preparation and the fuel oxidizer utilization techniques; in the case of good fuel and air preparation, the fuel hydrocarbon is theoretically converted into water vapour and carbon dioxide. At the same time, the fuel combustion may produce carbon monoxide and unburned hydrocarbon in the case of an incomplete combustion process. However, the carbon dioxide increases with improving the combustion conversion efficiency and reduces with burning lower carbon chain fuel.

**Figure 7:**
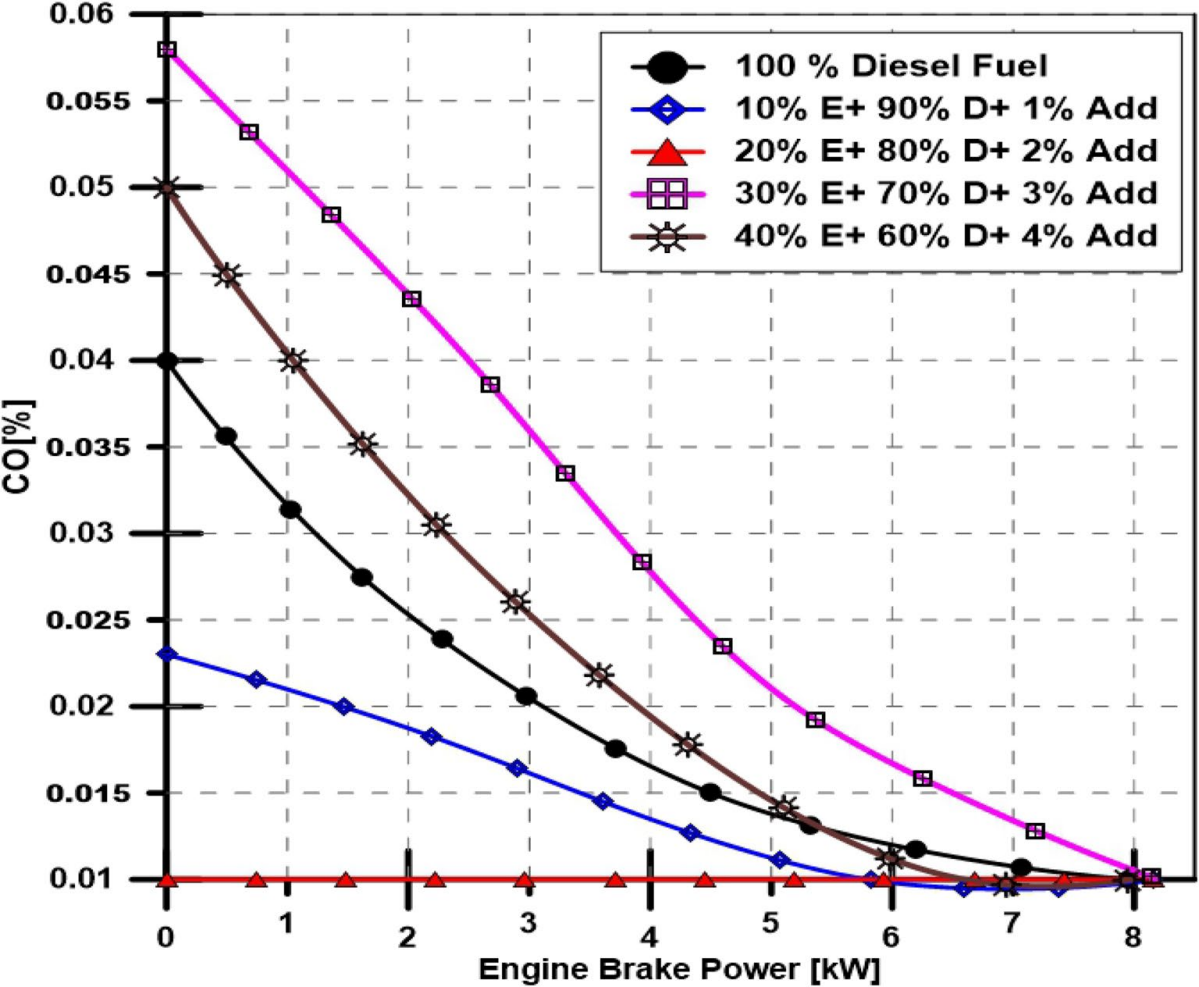
Effect of ethanol and TBP additives on CO emissions.

Results are shown in Fig. 7, which depicts Carbon monoxide emission percentages in the exhaust gas of the engine at various engine loads at a constant speed (1500 RPM). The results demonstrated that increasing the engine load decreased the percentage of CO emission concentration. Nevertheless, the ethanol dose and TBP additives percentages significantly impact the percentage of CO exhaust emissions. In comparing based fuel with blends fuel, 10 and 20% ethanol in the fuel blends (10% E + 90% D + 1% Add) and (20% E + 80% D + 2% Add) decreases the CO % than based fuel. While 30% and 40% ethanol in the fuel blend (30% E + 70% D + 3% Add) and (40% E + 60% D + 4% Add) increases the CO % more than based fuel. (20% E + 80% D + 2% Add) blend record the lowest CO % emissions. CO % ranged from 0.01% at high engine load to 0.058% at low.

#### Ethanol and TBP's impact on engine CO_2_ emission

**Figure 8:**
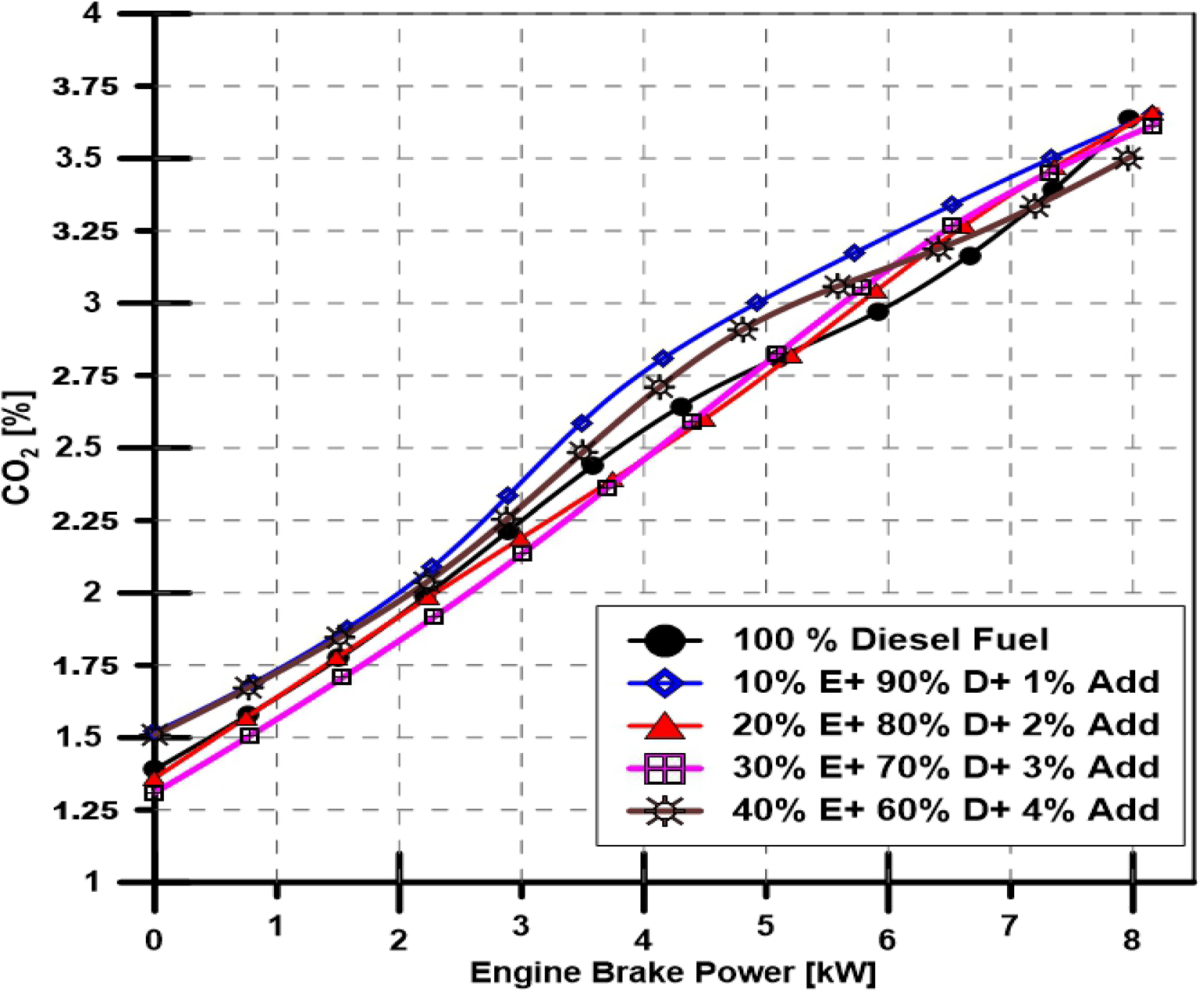
Effect of ethanol and TBP additives on CO_2_ emissions.

Results in Fig. 8 show the CO_2_% that exits in the engine's exhaust gases at various engine loads with variable ethanol and TBP additives in the fuel blend. However, as illustrated in Fig. 8, the linear relationship between the volume % of CO_2_ in the exhaust emission and engine load rose proportionately. With varying ethanol and TBP additives in the fuel blends, various engine loads, and a constant speed of 1500 PPM, those CO_2_ percentages vary unremarkably.

#### Ethanol and TBP's impact on engine HC emission

**Figure 9:**
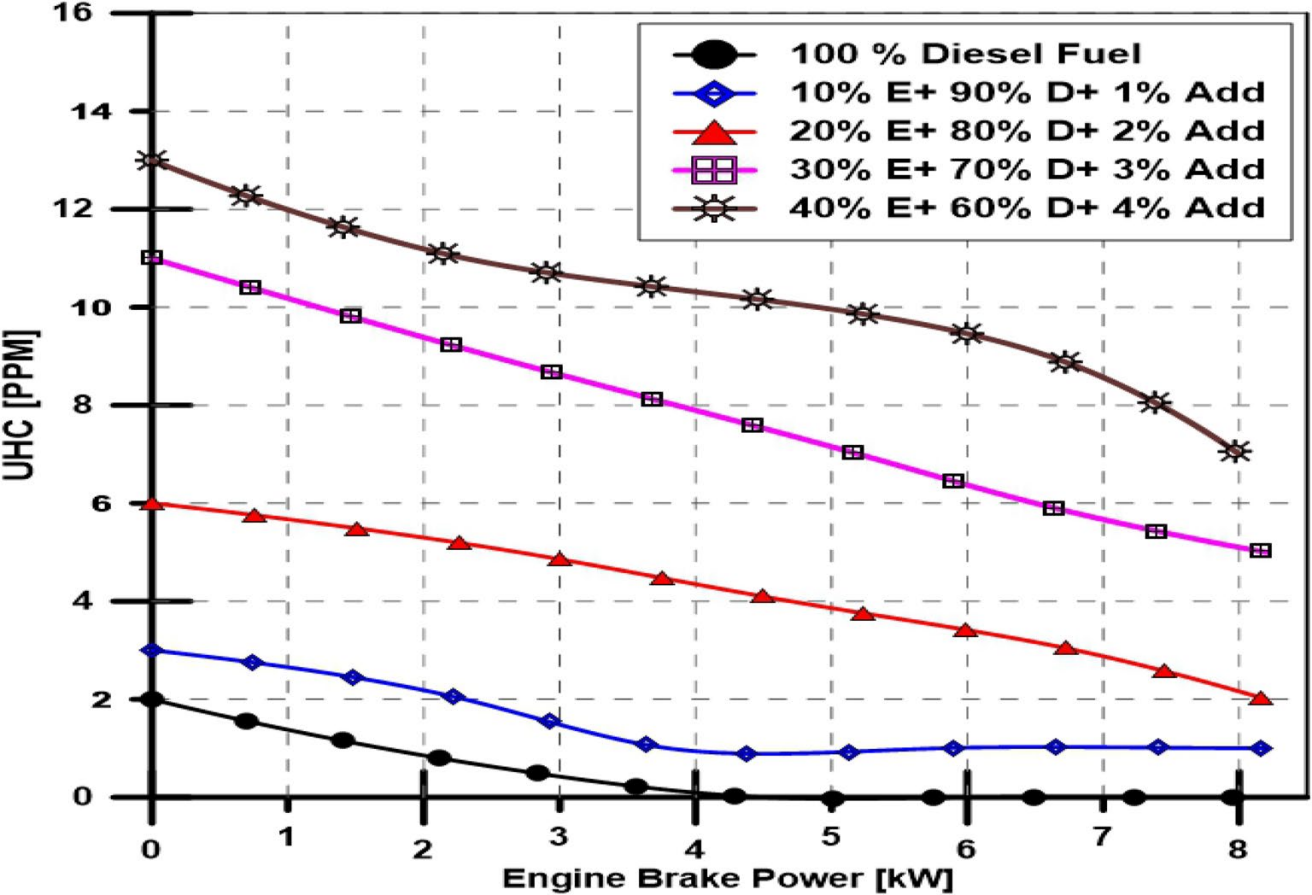
Effect of ethanol and TBP additives on unburned HC emissions.

Emissions of unburned hydrocarbon have been measured at various engine loads with ethanol doses ranging from 10 to 40% by volume and TBP additions from 1 to 4%. The results obtained are shown in Fig. 9. The base fuel, a 100% diesel fuel, was utilized to compare the effects of adding 10, 20, 30, and 40% ethanol and TBP increases from 1 to 4%, and it was found that the amount of UHCS increased as engine load increased. At the same time, the concentration of unburned hydrocarbons in the exhaust gases increased with the increasing ethanol dose.

#### Ethanol and TBP's impact on engine NOx emission

**Figure 10:**
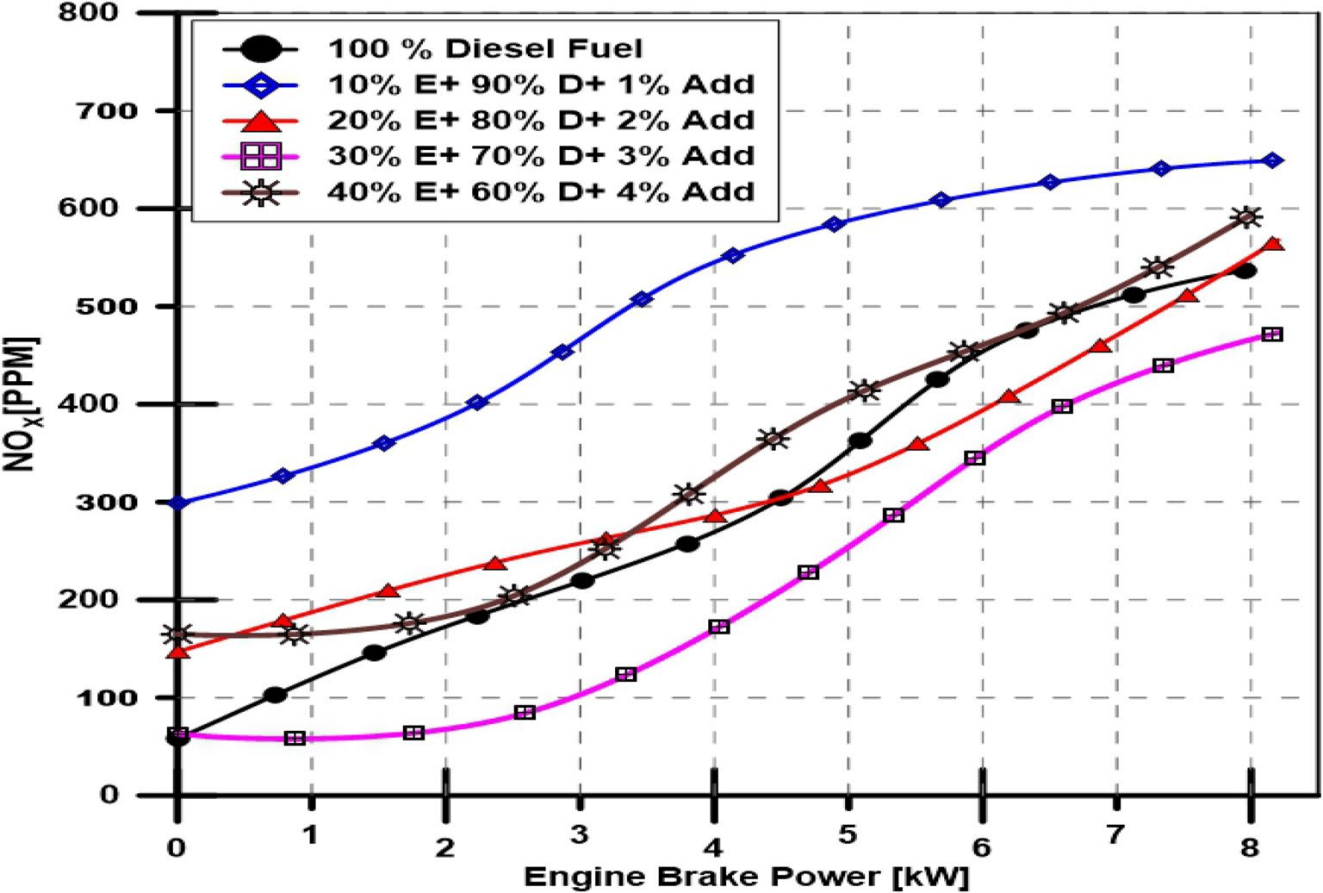
Effect of ethanol and TBP additives on NOx emissions.

The data in Fig. 10 illustrates the PPM value for nitrogen oxides in the engine exhaust gases for various doses of bioethanol in the fuel mixture at different engine loads. The NOx emissions for diesel fuel, the base engine fuel, increased exponentially as engine load increased. On the other hand, as the engine load varied, the NOx concentration levels went from 58 to 649 PPM.

#### Ethanol and TBP's impact on engine O_2_ emission

**Figure 11:**
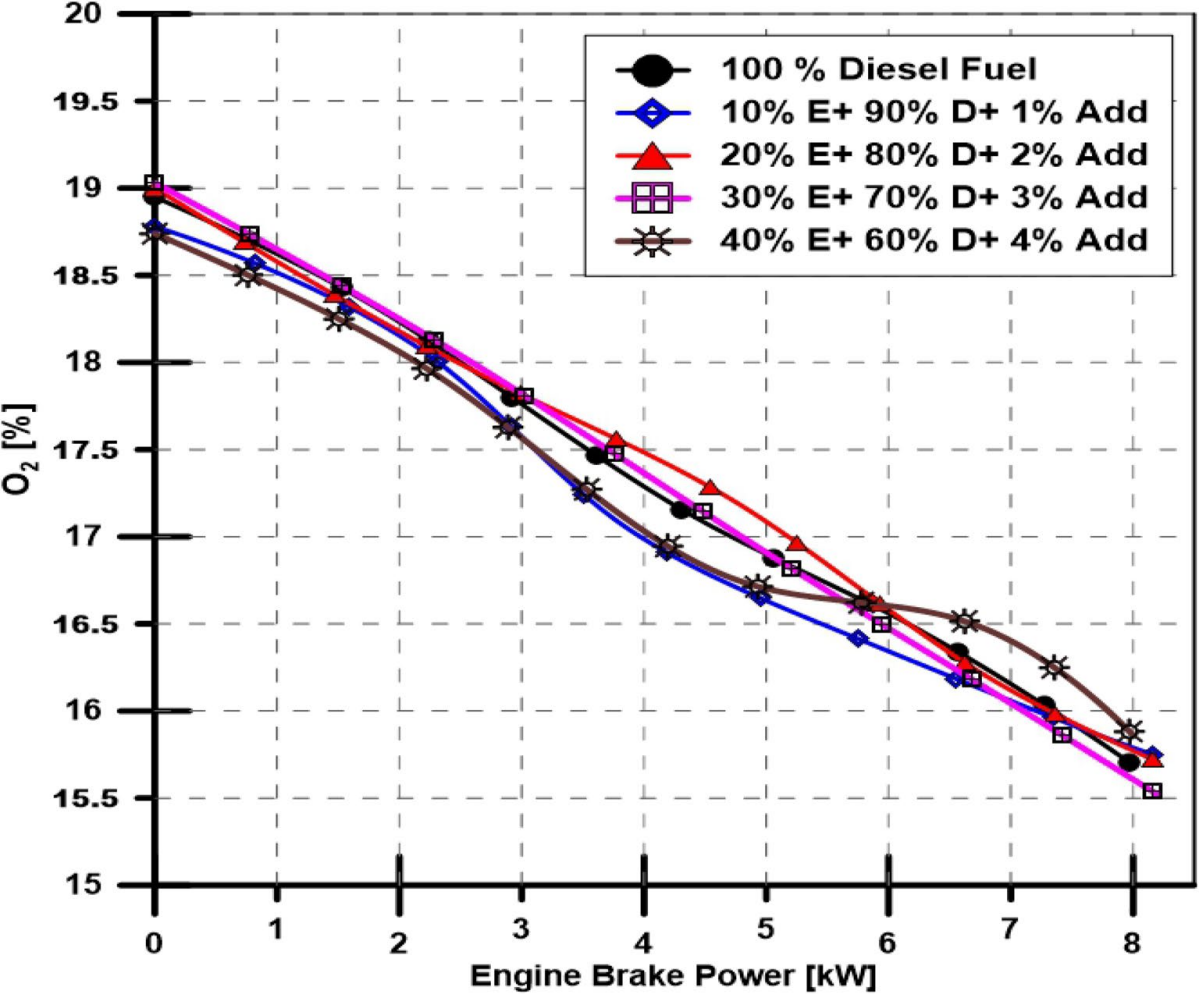
Effect of ethanol and TBP additives on O_2_ emissions.

As can be observed in Fig. 11, the acquired data showed a linear relationship between the engine load and the reduction in oxygen in the exhaust gases. As engine load increases, the oxygen percentage slightly rises compared to diesel fuel, the base engine fuel.

## Conclusions

The effects of varying the blends of diesel and ethanol fuel in amounts of 10%, 20%, 30%, and 40% by weight are determined through a lengthy experiment. It was discovered that, for the first time, adding TBP as a surfactant improved the stability of diesel fuel with high ethanol percentages. In the set of experiments, each of the biofuel blends is utilized with a diesel engine operating at 1500 rpm under various load conditions. Emissions were collected and evaluated by an exhaust gas analyzer system in each test. The exhaust gas temperature, the brake thermal efficiency of the engine (BTE), and brake-specific fuel consumption (BSFC) are all measured at different engine load conditions. When ethanol was increased to 40% in blends against diesel fuel (base fuel), the following conclusion was reached:

- BSFC and engine BTE show an ordinary change compared to base fuel.
- Slight drops in engine fumes gas temperature.
- When bioethanol is increased to 40% of the base volume, it causes an increase in the combustion of unburned hydrocarbons and the emission of CO.
- O_2_ emissions slightly increased while CO_2_ emissions decreased.

## Data Availability

The datasets generated during and analyzed during the current study are available from the second and corresponding author upon reasonable request.

## References

[1] Ayad SMME, Vago CL, Belchior CRP, Sodré JR (2021). Cylinder pressure based calibration model for engines using ethanol, hydrogen and natural gas as alternative fuels. Energy Rep..

[2] Iea I (2011). World Energy Outlook 2011.

[3] Demirbas A (2007). Progress and recent trends in biofuels. Prog. Energy Combust. Sci..

[4] Demirbas A (2008). Biofuels sources, biofuel policy, biofuel economy and global biofuel projections. Energy Convers. Manag..

[5] de Faria MMN, Bueno JPVM, Ayad SMME, Belchior CRP (2017). Thermodynamic simulation model for predicting the performance of spark ignition engines using biogas as fuel. Energy Convers. Manag..

[6] Elkelawy M, Alm-Eldin Bastawissi H, Esmaeil KK, Radwan AM, Panchal H, Sadasivuni KK, Ponnamma D, Walvekar R (2019). Experimental studies on the biodiesel production parameters optimization of sunflower and soybean oil mixture and DI engine combustion, performance, and emission analysis fueled with diesel/biodiesel blends. Fuel.

[7] Elkelawy M, Bastawissi HA-E, Esmaeil KK, Radwan AM, Panchal H, Sadasivuni KK, Suresh M, Israr M (2020). Maximization of biodiesel production from sunflower and soybean oils and prediction of diesel engine performance and emission characteristics through response surface methodology. Fuel.

[8] Shivapuji AM, Dasappa S (2017). Quasi dimensional numerical investigation of syngas fuelled engine operation: MBT operation and parametric sensitivity analysis. Appl. Therm. Eng..

[9] Simsek S, Uslu S, Simsek H (2022). Proportional impact prediction model of animal waste fat-derived biodiesel by ANN and RSM technique for diesel engine. Energy.

[10] Simsek S, Uslu S, Sahin M, Arlı F, Bilgic G (2021). Impact of a novel fuel additive containing boron and hydrogen on diesel engine performance and emissions. Energy Source Part A Recovery Util. Environ. Effects.

[11] Simsek S, Uslu S, Simsek H (2022). Assessment of the fuel recovery potential of cattle, sheep, and chicken waste fats in diesel engine. Int. J. Environ. Sci. Technol..

[12] Şimşek S, Samet U (2021). The effect of using amyl alcohol in a diesel engine on performance and emission parameters. Int. J. Automot. Sci. Technol..

[13] Şimşek S, Samet U (2021). Analysis of the effects of cetane improver addition to diesel on engine performance and emissions. Int. J. Automot. Eng. Technol..

[14] Şimşek S (2020). Increasing cetane number of the diesel fuel by fuel additives. Int. J. Automot. Sci. Technol..

[15] Simsek S, Uslu S, Simsek H (2022). Response surface methodology-based parameter optimization of single-cylinder diesel engine fueled with graphene oxide dosed sesame oil/diesel fuel blend. Energy AI.

[16] Simsek S, Uslu S (2020). Determination of a diesel engine operating parameters powered with canola, safflower and waste vegetable oil based biodiesel combination using response surface methodology (RSM). Fuel.

[17] Simsek S, Uslu S (2020). Investigation of the effects of biodiesel/2-ethylhexyl nitrate (EHN) fuel blends on diesel engine performance and emissions by response surface methodology (RSM). Fuel.

[18] Mofijur M, Rasul MG, Hyde J, Azad AK, Mamat R, Bhuiya MMK (2016). Role of biofuel and their binary (diesel–biodiesel) and ternary (ethanol–biodiesel–diesel) blends on internal combustion engines emission reduction. Renew. Sustain. Energy Rev..

[19] Khalife E, Tabatabaei M, Demirbas A, Aghbashlo M (2017). Impacts of additives on performance and emission characteristics of diesel engines during steady state operation. Prog. Energy Combust. Sci..

[20] Pradelle F, Braga SL, de Aguiar Martins ARF, Turkovics F, Pradelle RNC (2019). Experimental assessment of some key physicochemical properties of diesel-biodiesel-ethanol (DBE) blends for use in compression ignition engines. Fuel.

[21] Elkelawy M, Kabeel AE, El Shenawy EA, Panchal H, Elbanna A, Bastawissi HA-E, Sadasivuni KK (2020). Experimental investigation on the influences of acetone organic compound additives into the diesel/biodiesel mixture in CI engine. Sustain. Energy Technol. Assess..

[22] Elkelawy, M., Bastawissi, H., Sekar, S. C., Karuppasamy, K., Vedaraman, N., Sathiyamoorthy, K. & Sathyamurthy, R. Numerical and experimental investigation of ethyl alcohol as oxygenator on the combustion, performance, and emission characteristics of diesel/cotton seed oil blends in homogenous charge compression ignition engine, 0148-7191, SAE Technical Paper (2018).

[23] Venu H, Raju VD, Lingesan S, Soudagar MEM (2021). Influence of Al_2_O_3_nano additives in ternary fuel (diesel-biodiesel-ethanol) blends operated in a single cylinder diesel engine: Performance, combustion and emission characteristics. Energy.

[24] El-Sheekh MM, Bedaiwy MY, El-Nagar AA, ElKelawy M, Bastawissi HA-E (2022). Ethanol biofuel production and characteristics optimization from wheat straw hydrolysate: Performance and emission study of DI-diesel engine fueled with diesel/biodiesel/ethanol blends. Renew. Energy.

[25] Ozer S, Akcay M, Vural E (2021). Effects of liquefed petroleum gas use in a turbocharged stratified injection engine using ethanol/gasoline as pilot fuel. Therm. Sci..

[26] Ertugrul I, Ulkir O, Ozer S, Ozel S (2022). Analysis of thermal barrier coated pistons in the COMSOL and the effects of their use with water + ethanol doped biodiesel. Therm. Sci..

[27] Ozer S, Doğan B (2022). Thermodynamic analyzes in a compression ignition engine using fuel oil diesel fuel blends. Therm. Sci..

[28] Demir U, Çelebi S, Özer S (2023). Experimental investigation of the effect of fuel oil, graphene and HHO gas addition to diesel fuel on engine performance and exhaust emissions in a diesel engine. Int. J. Hydrog. Energy.

[29] Özer S, Demir U, Koçyiğit S (2023). Effect of using borax decahydrate as nanomaterials additive diesel fuel on diesel engine performance and emissions. Energy.

[30] Shahir SA, Masjuki HH, Kalam MA, Imran A, Fattah IMR, Sanjid A (2014). Feasibility of diesel–biodiesel–ethanol/bioethanol blend as existing CI engine fuel: An assessment of properties, material compatibility, safety and combustion. Renew. Sustain. Energy Rev..

[31] Jiang G, Li J, Zhao L, Meng T, Yu J, Wang H, Hu J, Xu B (2022). Insights into the deoiling efficiency of oil-based cuttings by surfactant-free microemulsions. J. Environ. Chem. Eng..

[32] Elkelawy M, Alm-Eldin Bastawissi H, El Shenawy EA, Taha M, Panchal H, Sadasivuni KK (2021). Study of performance, combustion, and emissions parameters of DI-diesel engine fueled with algae biodiesel/diesel/n-pentane blends. Energy Convers. Manag. X.

[33] Garcia M, Rodríguez I, Cela R (2007). Microwave-assisted extraction of organophosphate flame retardants and plasticizers from indoor dust samples. J. Chromatogr. A.

[34] Servis MJ, Clark AE (2019). Surfactant-enhanced heterogeneity of the aqueous interface drives water extraction into organic solvents. Phys. Chem. Chem. Phys..

[35] Schulz WW, Navratil JD (1984). Science and Technology of Tributyl Phosphate. Synthesis, Properties, Reactions and Analysis.

[36] Olsen J (2022). Reducing the fuel consumption of light aircraft. Int. J. Sustain. Aviat..

[37] Ning L, Duan Q, Chen Z, Kou H, Liu B, Yang B, Zeng K (2020). A comparative study on the combustion and emissions of a non-road common rail diesel engine fueled with primary alcohol fuels (methanol, ethanol, and n-butanol)/diesel dual fuel. Fuel.

[38] Sharma P, Chauhan NR, Saraswat M (2021). Competency of alcoholic fuels as diesel blends. Recent Trends Therm. Eng..

[39] Iempremjit K (2021). Combustion characteristics of ethanol with ignition improver in a small diesel engine during start-up. Princess Naradhiwas Univ. J..

[40] Preetika R, Mehta PS, Kaisare NS, Basavaraj MG (2019). Kinetic stability of surfactant stabilized water-in-diesel emulsion fuels. Fuel.

[41] Ecklund EE, Bechtold RL, Timbario TJ, McCallum PW (1984). State-of-the-art report on the use of alcohols in diesel engines. SAE Trans..

[42] Ezeji T, Qureshi N, Blaschek HP (2007). Production of acetone–butanol–ethanol (ABE) in a continuous flow bioreactor using degermed corn and *Clostridium beijerinckii*. Process Biochem..

[43] Vertes AA, Qureshi N, Yukawa H, Blaschek HP (2011). Biomass to Biofuels: Strategies for Global Industries.

[44] Yao M, Wang H, Zheng Z, Yue Y (2010). Experimental study of n-butanol additive and multi-injection on HD diesel engine performance and emissions. Fuel.

[45] Markov VA, Kamaltdinov VG, Zykov SA, Sa B (2019). Optimization of the composition of blended biodiesel fuels with additives of vegetable oils. Int. J. Energy Clean Environ..

[46] Huang J, Wang Y, Li S, Roskilly AP, Yu H, Li H (2009). Experimental investigation on the performance and emissions of a diesel engine fuelled with ethanol–diesel blends. Appl. Therm. Eng..

[47] El-din S, Etaiw H, Elkelawy M, Elziny I, Taha M, Veza I, Alm-Eldin Bastawissi H (2022). Effect of nanocomposite SCP1 additive to waste cooking oil biodiesel as fuel enhancer on diesel engine performance and emission characteristics. Sustain. Energy Technol. Assess..

[48] Elkelawy M, El Shenawy EA, Alm-Eldin Bastawissi H, Shams MM, Panchal H (2022). A comprehensive review on the effects of diesel/biofuel blends with nanofluid additives on compression ignition engine by response surface methodology. Energy Convers. Manag..

[49] Elkelawy M, Etaiw SE-DH, Alm-Eldin Bastawissi H, Ayad MI, Radwan AM, Dawood MM (2021). Diesel/ biodiesel/silver thiocyanate nanoparticles/hydrogen peroxide blends as new fuel for enhancement of performance, combustion, and emission characteristics of a diesel engine. Energy.

[50] Elkelawy M, El Shenawy EA, Almonem SKA, Nasef MH, Panchal H, Bastawissi HA-E, Sadasivuni KK, Choudhary AK, Sharma D, Khalid M (2021). Experimental study on combustion, performance, and emission behaviours of diesel/WCO biodiesel/cyclohexane blends in DI-CI engine. Process Saf. Environ. Prot..

[51] Elbanna AM, Xiaobei C, Can Y, Elkelawy M, Bastawissi HA-E (2022). A comparative study for the effect of different premixed charge ratios with conventional diesel engines on the performance, emissions, and vibrations of the engine block. Environ. Sci. Pollution Res..

